# 3-Methyl-1-propargylquinoxalin-2(1*H*)-one

**DOI:** 10.1107/S1600536809032498

**Published:** 2009-08-22

**Authors:** Hanane Benzeid, Youssef Ramli, Laure Vendier, El Mokhtar Essassi, Seik Weng Ng

**Affiliations:** a Laboratoire de Chimie Organique Hétérocyclique, Pôle de compétences Pharmacochimie, Université Mohammed V-Agdal, BP 1014 Avenue Ibn Batout, Rabat, Morocco; b Laboratoire de Chimie de Coordination, 205 Route de Narbonne, Toulouse Cedex 04, France; c Department of Chemistry, University of Malaya, 50603 Kuala Lumpur, Malaysia

## Abstract

The ten-membered fused ring of the title compound, C_12_H_10_N_2_O, is essentially planar in the two independent mol­ecules of the asymmetric unit (r.m.s. deviations = 0.012 and 0.015 Å).

## Related literature

For the crystal structure of 1-ethyl-3-methyl­quinoxalin-2(1*H*)-one, see: Benzeid *et al.* (2008 ▶). For the synthesis of the reactant 3-methyl-1*H*-quinoxalin-2-one, see: Nikolaenko & Munro (2004 ▶).
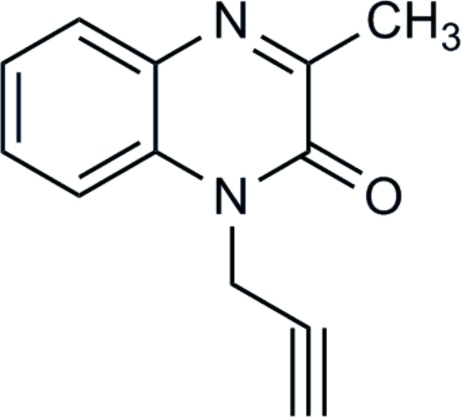


## Experimental

### 

#### Crystal data

C_12_H_10_N_2_O
*M* *_r_* = 198.22
Monoclinic, 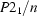

*a* = 21.124 (1) Å
*b* = 4.3709 (2) Å
*c* = 22.246 (1) Å
β = 105.354 (6)°
*V* = 1980.7 (2) Å^3^
*Z* = 8
Mo *K*α radiation
μ = 0.09 mm^−1^
*T* = 180 K
0.20 × 0.15 × 0.08 mm

#### Data collection

Oxford Diffraction Xcalibur diffractometer
Absorption correction: multi-scan (*CrysAlis RED*; Oxford Diffraction, 2006 ▶) *T*_min_ = 0.985, *T*_max_ = 0.991
14275 measured reflections
4058 independent reflections
2428 reflections with *I* > 2σ(*I*)
*R*_int_ = 0.046

#### Refinement

*R*[*F* ^2^ > 2σ(*F* ^2^)] = 0.036
*wR*(*F* ^2^) = 0.102
*S* = 0.97
4058 reflections
273 parameters
H-atom parameters constrained
Δρ_max_ = 0.19 e Å^−3^
Δρ_min_ = −0.22 e Å^−3^

### 

Data collection: *CrysAlis CCD* (Oxford Diffraction, 2006 ▶); cell refinement: *CrysAlis RED* (Oxford Diffraction, 2006 ▶); data reduction: *CrysAlis RED*; program(s) used to solve structure: *SHELXS97* (Sheldrick, 2008 ▶); program(s) used to refine structure: *SHELXL97* (Sheldrick, 2008 ▶); molecular graphics: *X-SEED* (Barbour, 2001 ▶); software used to prepare material for publication: *publCIF* (Westrip, 2009 ▶).

## Supplementary Material

Crystal structure: contains datablocks global, I. DOI: 10.1107/S1600536809032498/xu2590sup1.cif

Structure factors: contains datablocks I. DOI: 10.1107/S1600536809032498/xu2590Isup2.hkl

Additional supplementary materials:  crystallographic information; 3D view; checkCIF report

## Figures and Tables

**Fig. 1.**
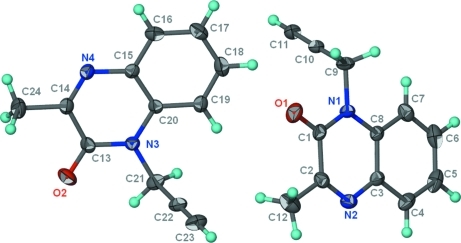
Thermal ellipsoid plot (Barbour, 2001) of the two independent molecules of C12H10N2O at the 70% probability level; hydrogen atoms are drawn as spheres of arbitrary radius.

## supplementary crystallographic information

### Experimental

To a solution of 3-methylquinoxalin-2(1*H*)-one (Nikolaenko & Munro *et al.*, 2004) (1 g, 6.22 mmol) in DMF (20 ml) was added propargyl bromide (0.82 ml, 6.22 mmol), potassium carbonate (1 g, 7.46 mmol) and a catalytic quantity of tetra-*n*-butylammonium bromide. The mixture was stirred at room temperature for 24 h. The solution was filtered and the solvent removed under reduced pressure. The residue was recrystallized from ethanol to afford 3-methyl-1-(propargyl)quinoxalin-2(1*H*)-one as colorless crystals.

### Refinement

Carbon-bound H-atoms were placed in calculated positions (C—H 0.95 to 0.99 Å) and were included in the refinement in the riding model approximation, with *U*(H) set to 1.2 to 1.5*U*(C).

### Crystal data

**Table:**

C_12_H_10_N_2_O	*F*(000) = 832
*M**_r_* = 198.22	*D*_x_ = 1.329 Mg m^−^^3^
Monoclinic, *P*2_1_/*n*	Mo *K*α radiation, λ = 0.71073 Å
Hall symbol: -P 2yn	Cell parameters from 5089 reflections
*a* = 21.124 (1) Å	θ = 2.7–32.2°
*b* = 4.3709 (2) Å	µ = 0.09 mm^−^^1^
*c* = 22.246 (1) Å	*T* = 180 K
β = 105.354 (6)°	Parallelepiped, yellow
*V* = 1980.7 (2) Å^3^	0.20 × 0.15 × 0.08 mm
*Z* = 8	

### Data collection

**Table:**

Oxford Diffraction Xcalibur diffractometer	4058 independent reflections
Radiation source: fine-focus sealed tube	2428 reflections with *I* > 2σ(*I*)
graphite	*R*_int_ = 0.046
ω and φ scans	θ_max_ = 26.4°, θ_min_ = 2.8°
Absorption correction: multi-scan (*CrysAlis RED*; Oxford Diffraction, 2006)	*h* = −22→26
*T*_min_ = 0.985, *T*_max_ = 0.991	*k* = −5→5
14275 measured reflections	*l* = −26→27

### Refinement

**Table:**

Refinement on *F*^2^	Primary atom site location: structure-invariant direct methods
Least-squares matrix: full	Secondary atom site location: difference Fourier map
*R*[*F*^2^ > 2σ(*F*^2^)] = 0.036	Hydrogen site location: inferred from neighbouring sites
*wR*(*F*^2^) = 0.102	H-atom parameters constrained
*S* = 0.97	*w* = 1/[σ^2^(*F*_o_^2^) + (0.0539*P*)^2^] where *P* = (*F*_o_^2^ + 2*F*_c_^2^)/3
4058 reflections	(Δ/σ)_max_ = 0.001
273 parameters	Δρ_max_ = 0.19 e Å^−^^3^
0 restraints	Δρ_min_ = −0.22 e Å^−^^3^

### Fractional atomic coordinates and isotropic or equivalent isotropic displacement parameters (Å^2^)

**Table:**

	*x*	*y*	*z*	*U*_iso_*/*U*_eq_	
O1	0.30129 (6)	0.4482 (3)	0.53550 (5)	0.0423 (3)	
O2	0.53899 (5)	0.4210 (3)	0.84138 (5)	0.0423 (3)	
N1	0.24291 (6)	0.1336 (3)	0.46002 (5)	0.0259 (3)	
N2	0.36089 (6)	−0.0417 (3)	0.43668 (6)	0.0301 (3)	
N3	0.45992 (6)	0.5883 (3)	0.75752 (6)	0.0248 (3)	
N4	0.40956 (6)	0.9390 (3)	0.83838 (6)	0.0299 (3)	
C1	0.30011 (8)	0.2584 (4)	0.49493 (7)	0.0284 (4)	
C2	0.36032 (8)	0.1492 (4)	0.48043 (7)	0.0299 (4)	
C3	0.30152 (8)	−0.1552 (4)	0.40141 (7)	0.0265 (4)	
C4	0.30203 (9)	−0.3586 (4)	0.35363 (7)	0.0359 (4)	
H4	0.3427	−0.4141	0.3460	0.043*	
C5	0.24506 (10)	−0.4804 (4)	0.31742 (8)	0.0438 (5)	
H5	0.2459	−0.6193	0.2848	0.053*	
C6	0.18630 (10)	−0.3990 (4)	0.32887 (8)	0.0441 (5)	
H6	0.1466	−0.4833	0.3038	0.053*	
C7	0.18398 (8)	−0.1987 (4)	0.37573 (8)	0.0342 (4)	
H7	0.1430	−0.1453	0.3830	0.041*	
C8	0.24165 (8)	−0.0749 (3)	0.41244 (7)	0.0250 (4)	
C9	0.18204 (8)	0.2356 (4)	0.47433 (8)	0.0324 (4)	
H9A	0.1884	0.4460	0.4914	0.039*	
H9B	0.1462	0.2416	0.4353	0.039*	
C10	0.16261 (7)	0.0368 (4)	0.51906 (7)	0.0285 (4)	
C11	0.14630 (8)	−0.1151 (4)	0.55593 (8)	0.0356 (4)	
H11	0.1331	−0.2379	0.5857	0.043*	
C12	0.42291 (8)	0.2734 (5)	0.52009 (9)	0.0484 (5)	
H12A	0.4594	0.2039	0.5040	0.073*	
H12B	0.4294	0.2006	0.5630	0.073*	
H12C	0.4212	0.4974	0.5194	0.073*	
C13	0.49034 (7)	0.5800 (4)	0.81993 (7)	0.0284 (4)	
C14	0.46119 (8)	0.7751 (4)	0.85929 (7)	0.0304 (4)	
C15	0.37878 (7)	0.9314 (4)	0.77527 (7)	0.0248 (4)	
C16	0.32214 (7)	1.1033 (4)	0.75256 (8)	0.0329 (4)	
H16	0.3056	1.2241	0.7805	0.039*	
C17	0.29004 (8)	1.1009 (4)	0.69069 (8)	0.0365 (4)	
H17	0.2514	1.2194	0.6756	0.044*	
C18	0.31410 (8)	0.9248 (4)	0.64995 (8)	0.0351 (4)	
H18	0.2914	0.9213	0.6070	0.042*	
C19	0.37008 (8)	0.7558 (4)	0.67064 (7)	0.0294 (4)	
H19	0.3863	0.6373	0.6422	0.035*	
C20	0.40306 (7)	0.7585 (3)	0.73351 (7)	0.0227 (3)	
C21	0.48885 (8)	0.4108 (4)	0.71564 (7)	0.0323 (4)	
H21A	0.5219	0.2677	0.7405	0.039*	
H21B	0.4541	0.2881	0.6872	0.039*	
C22	0.52007 (8)	0.6082 (4)	0.67891 (8)	0.0326 (4)	
C23	0.54466 (9)	0.7662 (5)	0.64907 (8)	0.0429 (5)	
H23	0.5646	0.8945	0.6248	0.051*	
C24	0.49583 (9)	0.7818 (5)	0.92679 (8)	0.0503 (5)	
H24A	0.5381	0.8860	0.9328	0.075*	
H24B	0.5032	0.5719	0.9426	0.075*	
H24C	0.4690	0.8918	0.9495	0.075*	

### Atomic displacement parameters (Å^2^)

**Table:**

	*U*^11^	*U*^22^	*U*^33^	*U*^12^	*U*^13^	*U*^23^
O1	0.0525 (8)	0.0439 (7)	0.0306 (7)	0.0029 (6)	0.0113 (6)	−0.0077 (6)
O2	0.0237 (6)	0.0535 (8)	0.0453 (7)	0.0048 (6)	0.0016 (5)	0.0074 (7)
N1	0.0248 (7)	0.0277 (7)	0.0275 (7)	0.0052 (6)	0.0108 (6)	0.0035 (6)
N2	0.0285 (8)	0.0303 (7)	0.0341 (8)	0.0050 (7)	0.0130 (6)	0.0079 (7)
N3	0.0218 (7)	0.0273 (7)	0.0259 (7)	−0.0017 (6)	0.0076 (6)	−0.0022 (6)
N4	0.0257 (8)	0.0407 (8)	0.0237 (7)	−0.0059 (7)	0.0074 (6)	−0.0041 (7)
C1	0.0349 (10)	0.0295 (9)	0.0216 (9)	0.0026 (8)	0.0089 (7)	0.0055 (8)
C2	0.0287 (9)	0.0295 (9)	0.0304 (9)	0.0024 (8)	0.0056 (7)	0.0078 (8)
C3	0.0319 (9)	0.0251 (8)	0.0248 (9)	0.0068 (8)	0.0118 (7)	0.0082 (7)
C4	0.0519 (12)	0.0287 (9)	0.0323 (10)	0.0102 (9)	0.0202 (9)	0.0063 (8)
C5	0.0722 (15)	0.0324 (10)	0.0271 (10)	0.0005 (10)	0.0133 (10)	−0.0017 (8)
C6	0.0542 (13)	0.0368 (11)	0.0324 (10)	−0.0058 (10)	−0.0041 (9)	0.0046 (9)
C7	0.0323 (10)	0.0330 (10)	0.0341 (10)	0.0008 (8)	0.0035 (8)	0.0089 (8)
C8	0.0309 (9)	0.0223 (8)	0.0228 (8)	0.0040 (8)	0.0089 (7)	0.0061 (7)
C9	0.0301 (9)	0.0324 (9)	0.0394 (10)	0.0100 (8)	0.0173 (8)	0.0052 (8)
C10	0.0227 (8)	0.0355 (9)	0.0285 (9)	0.0043 (8)	0.0086 (7)	−0.0034 (8)
C11	0.0274 (9)	0.0492 (11)	0.0323 (10)	0.0023 (9)	0.0115 (8)	0.0025 (9)
C12	0.0335 (11)	0.0510 (12)	0.0554 (12)	−0.0012 (10)	0.0023 (9)	0.0028 (10)
C13	0.0192 (8)	0.0364 (10)	0.0276 (9)	−0.0079 (8)	0.0028 (7)	0.0023 (8)
C14	0.0250 (9)	0.0424 (10)	0.0236 (9)	−0.0082 (8)	0.0064 (7)	−0.0001 (8)
C15	0.0211 (8)	0.0293 (8)	0.0251 (9)	−0.0075 (7)	0.0083 (7)	−0.0020 (7)
C16	0.0255 (9)	0.0325 (10)	0.0429 (11)	−0.0003 (8)	0.0130 (8)	−0.0012 (9)
C17	0.0232 (9)	0.0396 (11)	0.0440 (11)	0.0002 (8)	0.0041 (8)	0.0103 (9)
C18	0.0316 (10)	0.0416 (10)	0.0286 (9)	−0.0087 (9)	0.0017 (8)	0.0076 (9)
C19	0.0289 (9)	0.0354 (9)	0.0237 (9)	−0.0068 (8)	0.0067 (7)	−0.0012 (8)
C20	0.0177 (8)	0.0248 (8)	0.0253 (8)	−0.0055 (7)	0.0052 (6)	0.0010 (7)
C21	0.0355 (9)	0.0298 (9)	0.0347 (9)	0.0001 (8)	0.0144 (8)	−0.0050 (8)
C22	0.0273 (9)	0.0364 (10)	0.0374 (10)	0.0010 (8)	0.0144 (8)	−0.0074 (8)
C23	0.0389 (11)	0.0477 (11)	0.0493 (11)	0.0020 (9)	0.0244 (9)	−0.0007 (10)
C24	0.0372 (11)	0.0816 (15)	0.0279 (10)	−0.0060 (11)	0.0014 (8)	−0.0031 (10)

### Geometric parameters (Å, °)

**Table:**

O1—C1	1.2213 (18)	C9—H9B	0.9900
O2—C13	1.2277 (18)	C10—C11	1.176 (2)
N1—C1	1.365 (2)	C11—H11	0.9500
N1—C8	1.3917 (19)	C12—H12A	0.9800
N1—C9	1.4732 (19)	C12—H12B	0.9800
N2—C2	1.285 (2)	C12—H12C	0.9800
N2—C3	1.383 (2)	C13—C14	1.469 (2)
N3—C13	1.3673 (19)	C14—C24	1.486 (2)
N3—C20	1.3934 (19)	C15—C16	1.390 (2)
N3—C21	1.4643 (19)	C15—C20	1.396 (2)
N4—C14	1.285 (2)	C16—C17	1.364 (2)
N4—C15	1.3826 (18)	C16—H16	0.9500
C1—C2	1.472 (2)	C17—C18	1.384 (2)
C2—C12	1.484 (2)	C17—H17	0.9500
C3—C4	1.388 (2)	C18—C19	1.367 (2)
C3—C8	1.395 (2)	C18—H18	0.9500
C4—C5	1.367 (2)	C19—C20	1.388 (2)
C4—H4	0.9500	C19—H19	0.9500
C5—C6	1.378 (3)	C21—C22	1.460 (2)
C5—H5	0.9500	C21—H21A	0.9900
C6—C7	1.372 (2)	C21—H21B	0.9900
C6—H6	0.9500	C22—C23	1.171 (2)
C7—C8	1.385 (2)	C23—H23	0.9500
C7—H7	0.9500	C24—H24A	0.9800
C9—C10	1.459 (2)	C24—H24B	0.9800
C9—H9A	0.9900	C24—H24C	0.9800
			
C1—N1—C8	122.01 (13)	H12A—C12—H12B	109.5
C1—N1—C9	116.60 (13)	C2—C12—H12C	109.5
C8—N1—C9	121.38 (13)	H12A—C12—H12C	109.5
C2—N2—C3	118.28 (14)	H12B—C12—H12C	109.5
C13—N3—C20	121.95 (13)	O2—C13—N3	121.98 (15)
C13—N3—C21	117.96 (13)	O2—C13—C14	122.47 (15)
C20—N3—C21	120.10 (13)	N3—C13—C14	115.55 (14)
C14—N4—C15	118.69 (14)	N4—C14—C13	123.89 (15)
O1—C1—N1	122.25 (15)	N4—C14—C24	119.90 (16)
O1—C1—C2	122.16 (16)	C13—C14—C24	116.20 (15)
N1—C1—C2	115.59 (14)	N4—C15—C16	118.96 (14)
N2—C2—C1	123.96 (15)	N4—C15—C20	122.13 (14)
N2—C2—C12	120.14 (15)	C16—C15—C20	118.91 (14)
C1—C2—C12	115.90 (15)	C17—C16—C15	120.85 (16)
N2—C3—C4	118.32 (15)	C17—C16—H16	119.6
N2—C3—C8	122.53 (14)	C15—C16—H16	119.6
C4—C3—C8	119.15 (16)	C16—C17—C18	119.63 (16)
C5—C4—C3	121.12 (17)	C16—C17—H17	120.2
C5—C4—H4	119.4	C18—C17—H17	120.2
C3—C4—H4	119.4	C19—C18—C17	120.99 (16)
C4—C5—C6	119.08 (17)	C19—C18—H18	119.5
C4—C5—H5	120.5	C17—C18—H18	119.5
C6—C5—H5	120.5	C18—C19—C20	119.59 (16)
C7—C6—C5	121.35 (17)	C18—C19—H19	120.2
C7—C6—H6	119.3	C20—C19—H19	120.2
C5—C6—H6	119.3	C19—C20—N3	122.26 (14)
C6—C7—C8	119.64 (17)	C19—C20—C15	120.01 (14)
C6—C7—H7	120.2	N3—C20—C15	117.72 (13)
C8—C7—H7	120.2	C22—C21—N3	111.69 (13)
C7—C8—N1	122.76 (14)	C22—C21—H21A	109.3
C7—C8—C3	119.66 (15)	N3—C21—H21A	109.3
N1—C8—C3	117.57 (14)	C22—C21—H21B	109.3
C10—C9—N1	112.71 (13)	N3—C21—H21B	109.3
C10—C9—H9A	109.1	H21A—C21—H21B	107.9
N1—C9—H9A	109.1	C23—C22—C21	179.5 (2)
C10—C9—H9B	109.1	C22—C23—H23	180.0
N1—C9—H9B	109.1	C14—C24—H24A	109.5
H9A—C9—H9B	107.8	C14—C24—H24B	109.5
C11—C10—C9	177.82 (17)	H24A—C24—H24B	109.5
C10—C11—H11	180.0	C14—C24—H24C	109.5
C2—C12—H12A	109.5	H24A—C24—H24C	109.5
C2—C12—H12B	109.5	H24B—C24—H24C	109.5
			
C8—N1—C1—O1	177.78 (14)	C20—N3—C13—O2	177.17 (14)
C9—N1—C1—O1	−1.0 (2)	C21—N3—C13—O2	−2.8 (2)
C8—N1—C1—C2	−2.2 (2)	C20—N3—C13—C14	−3.2 (2)
C9—N1—C1—C2	179.07 (12)	C21—N3—C13—C14	176.86 (13)
C3—N2—C2—C1	−0.8 (2)	C15—N4—C14—C13	0.6 (2)
C3—N2—C2—C12	179.06 (15)	C15—N4—C14—C24	179.19 (15)
O1—C1—C2—N2	−177.42 (15)	O2—C13—C14—N4	−178.51 (16)
N1—C1—C2—N2	2.6 (2)	N3—C13—C14—N4	1.9 (2)
O1—C1—C2—C12	2.7 (2)	O2—C13—C14—C24	2.9 (2)
N1—C1—C2—C12	−177.34 (14)	N3—C13—C14—C24	−176.77 (15)
C2—N2—C3—C4	179.21 (13)	C14—N4—C15—C16	178.69 (15)
C2—N2—C3—C8	−1.3 (2)	C14—N4—C15—C20	−1.9 (2)
N2—C3—C4—C5	179.55 (15)	N4—C15—C16—C17	−179.62 (15)
C8—C3—C4—C5	0.0 (2)	C20—C15—C16—C17	0.9 (2)
C3—C4—C5—C6	−0.1 (2)	C15—C16—C17—C18	0.1 (3)
C4—C5—C6—C7	0.0 (3)	C16—C17—C18—C19	−0.8 (3)
C5—C6—C7—C8	0.0 (2)	C17—C18—C19—C20	0.6 (2)
C6—C7—C8—N1	178.84 (14)	C18—C19—C20—N3	179.23 (14)
C6—C7—C8—C3	0.0 (2)	C18—C19—C20—C15	0.4 (2)
C1—N1—C8—C7	−178.59 (15)	C13—N3—C20—C19	−176.69 (14)
C9—N1—C8—C7	0.1 (2)	C21—N3—C20—C19	3.2 (2)
C1—N1—C8—C3	0.3 (2)	C13—N3—C20—C15	2.1 (2)
C9—N1—C8—C3	178.99 (13)	C21—N3—C20—C15	−177.94 (13)
N2—C3—C8—C7	−179.48 (14)	N4—C15—C20—C19	179.38 (14)
C4—C3—C8—C7	0.0 (2)	C16—C15—C20—C19	−1.2 (2)
N2—C3—C8—N1	1.6 (2)	N4—C15—C20—N3	0.5 (2)
C4—C3—C8—N1	−178.93 (13)	C16—C15—C20—N3	180.00 (14)
C1—N1—C9—C10	−93.04 (17)	C13—N3—C21—C22	−107.74 (16)
C8—N1—C9—C10	88.22 (17)	C20—N3—C21—C22	72.32 (18)
